# A prospective investigation of rumination and executive control in predicting overgeneral autobiographical memory in adolescence

**DOI:** 10.3758/s13421-017-0779-z

**Published:** 2018-01-16

**Authors:** Tracy M. Stewart, Simon C. Hunter, Sinéad M. Rhodes

**Affiliations:** 1 Moray House School of Education, University of Edinburgh, Edinburgh, EH8 8AQ Scotland UK; 2 School of Psychological Sciences and Health, University of Strathclyde, Glasgow, G1 1QE Scotland UK; 3 SMC Research Centre for Learning Difficulties, Child Life & Health, Centre for Clinical Brain Sciences, University of Edinburgh, 20 Sylvan Place, Edinburgh, EH9 1UW UK

**Keywords:** Overgeneral autobiographical memory, Adolescence, Rumination, Executive control, Emotion

## Abstract

The CaR-FA-X model (Williams et al., 2007), or *c*apture and *r*umination (CaR), *f*unctional *a*voidance (FA), and impaired e*x*ecutive control (X), is a model of overgeneral autobiographical memory (OGM). Two mechanisms of the model, rumination and executive control, were examined in isolation and in interaction in order to investigate OGM over time. Across two time points, six months apart, a total of 149 adolescents (13–16 years) completed the minimal-instruction autobiographical memory test, a measure of executive control with both emotional and nonemotional stimuli, and measures of brooding rumination and reflective pondering. The results showed that executive control for emotional information was negatively associated with OGM, but only when reflective pondering levels were high. Therefore, in the context of higher levels of reflective pondering, greater switch costs (i.e., lower executive control) when processing emotional information predicted a decrease in OGM over time.

Autobiographical memory (AM) is a type of episodic memory storage system for past personal experiences and semantic information about the self (Conway & Pleydell-Pearce, 2000). The structure of AM is hierarchical, forming a broad life story of past personal memories from the highest level of memory representation for prolonged periods of time, to general memories for single and repeated events, to event specific memory representations (*self-memory* model; Conway & Pleydell-Pearce, 2000). The ability to retrieve specific memories, defined as a memory for a personally experienced event that occurred at a particular time and place and lasted less than a day, has a number of important functions. For example, remembering specific details from past events can aid problem solving, guide future behaviors, and help regulate emotions (Raes, Hermans, de Decker, Eelen, & Williams, 2003). Unlike specific memories, remembering events in an overgeneral, nonspecific way has consistently been associated with emotional difficulties. Remembering events in such a nonspecific, overgeneral way refers to the phenomenon of *overgeneral autobiographical memory* (OGM). There are two forms of OGM: categorical memories, defined as memories for categories of events (e.g., every weekend when I visit my grandparents), and extended memories, defined as general events that last over an extended time frame (e.g., my summer holiday in Spain).

The association between OGM and depression is well-documented, and OGM is associated with the onset, diagnosis, and course of depression in adults (Kaviani, Rahimi, Rahimi-Darabad, & Naghavi, 2011; Sumner, Griffith, & Mineka, 2010; Williams et al., 2007), as well as with the onset of depression in adolescence (Kleim & Ehlers, 2008). OGM is a stable characteristic in adults with depression, those recovered from depression, and those at risk of depression (Brittlebank, Scott, Williams, & Ferrier, 1993; Mackinger, Pachinger, Leibetseder, & Fartacek, 2000). Furthermore, Williams and Dritschel (1992) reported that group differences in OGM between suicidal patients and controls were due to the retrieval of categoric memories, with no differences in extended memories. In a recent review of the adult literature, Sumner et al. (2010) reported that OGM was associated with symptoms of depression and predicted increased symptoms of depression over and above initial symptoms. Fewer studies have investigated the phenomenon of OGM with child and adolescent populations. Within the limited number that have, OGM (for categoric, categoric and extended memories combined, and reduced specific memories) has been shown to predict later depressive symptoms and depressive disorder (Hitchcock, Nixon, & Weber, 2014b), even after controlling for age and baseline OGM, depressive symptoms, and IQ (Rawal & Rice, 2012a). Furthermore, OGM is a stable characteristic in both adolescents and adults who are recovered from depression (Kuyken & Dalgleish, 2011; Mackinger et al., 2000). Given the significance of OGM in understanding adolescent depression, an important research objective is to investigate the theoretical underpinnings of OGM, particularly during the developmental period associated with the onset of depressive symptoms (Dekker et al., 2007) and disorder (Kessler et al., 2005). A greater understanding of OGM in adolescence could lead to the creation of focused techniques designed to reduce the risk associated with it.

The CaR-FA-X model (Williams et al., 2007; see Fig. 1 for a visualization) is based on Conway and Pleydell-Pearce’s (2000) self-memory model, which posits that the search for a specific memory requires a hierarchical search through the autobiographical memory knowledge base, which has three levels of memory descriptions. The broadest, top level of the knowledge base holds memories for events of prolonged time periods (e.g., my time at university). Below these, midlevel knowledge includes more general memories for single or repeat events (e.g., my summer holiday in Spain or every day on the train to work). Finally, the lowest level of the knowledge base relates to event-specific knowledge that primarily consists of summary records of sensory–perceptual processing during an event (e.g., my 30th birthday party last Saturday). Conway and Pleydell-Pearce stated that memories for events can be recalled in two ways, either through a generative search process or by direct retrieval. *Generative retrieval* refers to top-down processing that spreads down the AM knowledge base, activating broad memory representations, then general memories, and finally specific memories. The CaR-FA-X model (Williams et al., 2007) is the most prominent and comprehensive theory of OGM and is a framework for generative retrieval. The model posits that due to difficulties in one or more of the three mechanisms (i.e., CaR: *c*apture and *r*umination; FA: *f*unctional *a*voidance; and/or X: impaired e*x*ecutive control), the generative search is disrupted early and thus truncated, leading in turn to OGM. The different mechanisms can work in isolation or in interaction (Williams et al., 2007).

**Fig. 1:**
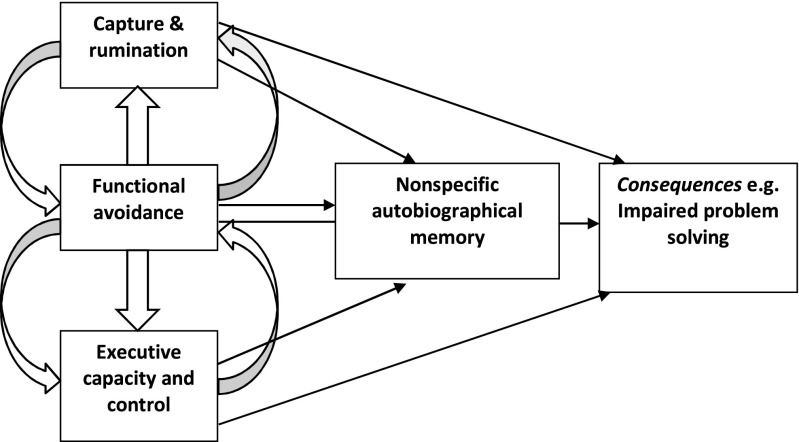
The CaR-FA-X model. Three processes contributing to overgeneral memory—capture and rumination (CaR), functional avoidance (FA), and impaired executive capacity and control (X)—can each have effects on cognition and behavior (e.g., problem solving), either independently or through their individual or combined effects on autobiographical memory. From “Autobiographical Memory Specificity and Emotional Disorder,” by J. M. G. Williams, T. Barnhofer, C. Crane, D. Hermans, F. Raes, E. Watkins, & T. Dalgleish, 2007, *Psychological Bulletin*, *133*, p. 122–148. Copyright 2007 by the name American Psychological Association. Reprinted with permission

The *capture and rumination* mechanism proposes that attention can be captured by self-relevant conceptual, abstract information activated at an early stage of retrieval. This can then activate ruminative thinking (i.e., persistent and recurring focus on negative thoughts, depressive affect, and their consequences; Nolen-Hoeksema, 1991), resulting in movement from categoric to other categoric memories instead of down the hierarchy to a specific memory; hence, the search for a specific memory is truncated. The second mechanism of the CaR-FA-X model is *functional avoidance*, which refers to the interruption of the memory search at a general level, typically in trauma-exposed populations. This is a method of affect regulation, since general memories are thought to result in less affect than specific memories. This mechanism is similar to those proposed in previous theories of OGM, where OGM was a strategy used by traumatized children as a way of avoiding either the negative affect associated with specific memories (Williams, 1996; affect regulation theory) or the sensory–perceptual elements of specific memories of traumatic events (Conway & Pleydell-Pearce, 2000; the self-memory model). These earlier theories of OGM suggest that avoidance is a key factor in OGM, and the CaR-FA-X model (Williams et al., 2007) builds on these previous theories by recognizing that a number of factors, including functional avoidance, contribute to OGM in both trauma-exposed and non-trauma-exposed populations (Williams et al., 2007).

The third and final mechanism is *impaired executive control* (sometimes referred to as *cognitive control* or *executive functions*). Executive control refers to a collection of cognitive processes responsible for the control, coordination, and flexibility of cognitive processes toward goal-directed behavior (Roberts, 1998; Shah & Miyake, 1999; Williams et al., 2007). There is ongoing debate concerning the nature and components of executive control (Diamond, 2013), but three separable but related executive functions have been proposed: inhibition, working memory updating, and switching (Miyake et al., 2000). Recently, Diamond proposed that higher-order executive functions, such as planning, problem solving, and reasoning skills, are built upon these initial executive functions. There are a number of cognitive theories of OGM. One group of theories is the resource allocation theories, which postulate a limit on the amount of cognitive capacity an individual has available, and that engaging in cognitive processing can lead to deficits in other cognitive resources (Ellis & Ashbrook, 1988). For example, cognitive processes such as rumination (the suppression of other thoughts or intrusions) may use up cognitive capacity that could otherwise have been allocated to the search for a specific memory (see Moore & Zoellner, 2007; van Vreeswijk & de Wilde, 2004). The CaR-FA-X model (Williams et al., 2007) posits that impairment in executive control can hamper a person’s ability to successfully search for and retrieve specific memories. For example, difficulties in the ability to inhibit irrelevant information and switch between, update, and hold information in working memory may truncate the search for a specific memory (Sumner et al., 2010; Williams et al., 2007).

The CaR-FA-X model (Williams et al., 2007) was developed to characterize OGM in clinical populations. Since Williams et al. (2007) formulated this theory, evidence has indicated that capture and rumination problems, as well as difficulties in executive control, contribute to OGM in nonclinical populations (Sumner, Griffith, & Mineka, 2011). A review of the literature with mainly adult populations (Sumner, 2012) concluded that the CaR-FA-X model is an important contribution to our understanding of OGM in both clinical and nonclinical populations. More recently, a review of the CaR-FA-X model within the child and adolescent literatures has supported the utility of the model in both clinical and nonclinical populations, while noting the need for further research in this area (see Stewart, Hunter, & Rhodes, 2017). Research investigating the CaR-FA-X model in child and adolescent populations has produced mixed findings. Capture errors have been associated with OGM in nonclinical, mixed-gender, community adolescent populations (Schoofs, Hermans, & Raes, 2012) as well as in subclinical (self-reported symptoms of depression) populations of mixed-gender trauma-exposed (physical and sexual abuse) and nonmaltreated children and adolescents (Valentino, Toth, & Cicchetti, 2009). However, rumination has only been supported in one study, which utilized a clinical adolescent population (Park, Goodyer, & Teasdale, 2004). Park, Goodyer and Teasdale examined OGM both pre and post an experimental rumination-and-distraction manipulation task, with samples of depressed adolescents and partially remitted adolescents, as compared with a psychiatric control group and a community control sample. In contrast to distraction, rumination resulted in increased OGM in the group diagnosed with depression (full and partially remitted). These findings suggest that rumination in isolation is not associated with OGM in nonclinical populations but may be associated with OGM in the context of clinical depression.

Research with nonclinical adolescents at familial risk of depression suggests that rumination in isolation does not predict OGM but that rumination in interaction with executive functioning (albeit assessed with a very general measure of executive functioning, the block design task, which assesses visuo-constructional ability) does predict OGM at a one-year follow-up (Rawal & Rice, 2012b). This suggests that identifying interactions between rumination and executive control may be more effective in explaining levels of OGM than examining the mechanisms in isolation. Indeed, research that investigates OGM and executive control in isolation has produced mixed findings. For example, although OGM has been associated with difficulties in inhibitory processing (Raes, Verstraeten, Bijttebier, Vasey, & Dalgleish, 2010) and category fluency (Valentino, Bridgett, Hayden, & Nuttall, 2012), other researchers have reported no such association between OGM and inhibition, switching, letter fluency (Valentino et al., 2012), or working memory (de Decker, Hermans, Raes, & Eelen, 2003).

The lack of empirical support for the executive control mechanism may be attributed to the paucity of studies that have employed emotional tasks of executive control. This may be problematic, since a growing literature has shown that executive control impairments appear to be more pronounced when processing emotional information (Joormann & Gotlib, 2010). Similarly, because rumination, particularly brooding rumination, involves a passive attentional focus on the meaning of negative emotions and thoughts (Treynor, Gonzalez, & Nolen-Hoeksema, 2003), the association between rumination and executive control may be more prominent when cognitive tasks use emotional rather than neutral stimuli.

It is also important to note that few studies have distinguished between the two subcomponents of rumination, reflective pondering and brooding rumination. *Reflective pondering*, defined as a nonjudgmental attentional focus on problem solving, is an adaptive form of rumination that is negatively associated with symptoms of depression (Arditte & Joormann, 2011). *Brooding rumination*, defined as a maladaptive component of rumination with a passive attentional focus on the meaning of negative and self-blaming thoughts (Treynor et al., 2003), has in contrast been positively associated with major depressive disorder and depressive symptoms in adolescence (Burwell & Shirk, 2007; Gibb, Grassia, Stone, Uhrlass, & McGreary, 2012). It is therefore important to consider the distinction between these two forms of rumination, but the literature on OGM in adolescence has seldom done so. Schoofs, Hermans, and Raes (2012), in two studies, did not find any associations between either brooding rumination or reflective pondering and OGM. However, Schoofs et al. did not consider whether there was an interaction between executive control and either form of rumination in OGM. In light of Rawal and Rice’s (2012b) finding concerning general rumination, we propose that the effects of executive control may be moderated by rumination, and the present study is the first to investigate this possibility.

To best characterize and understand the development of OGM, longitudinal studies are required. Two studies have prospectively investigated the interacting effects of executive control and rumination on OGM in child and adolescent populations. Hitchcock, Nixon, and Weber (2014a) investigated the effects of rumination on OGM across a 6-month period but found no main effect nor any interaction between rumination and impairment in executive control (working memory capacity, working memory updating, verbal fluency, or inhibition). This finding is in contrast to Rawal and Rice (2012b), who found that rumination in the context of low executive control (visuo-constructional ability) predicted reduced autobiographical memory specificity at a one-year follow-up (Rawal & Rice, 2012b). These two studies may have reached different results because they involved samples drawn from different populations: Hitchcock et al. (2014a) tested nonclinical preadolescents, whereas Rawal and Rice (2012b) tested adolescents who had a parent with a history of recurrent depression. The age of the participants may also have been a contributing factor here, since Rawal and Rice (2012b) recruited an adolescent sample (*M* = 13.64 years), whereas Hitchcock et al.’s (2014a) sample were preadolescents (*M* = 11.90 years). Rumination is relatively unstable in childhood (Driscoll, 2004) and only becomes a trait-like, more stable predictor of depression in adolescence (Rood, Roelofs, Bogels, Nolen-Hoeksema, & Schouten, 2009). Therefore, rumination may not yet have developed enough or been present long enough in the preadolescent sample from the Hitchcock et al. (2014a) study to exert an effect of executive control on OGM. This suggests that to examine the relation between rumination and executive control in predicting OGM, an adolescent sample over the age of 13 years may be necessary.

It should also be noted that executive control was assessed in different ways in the Hitchcock et al. (2014a) and Rawal and Rice (2012b) studies. Hitchcock et al. (2014a) referred to executive control as “a score” on their measures of executive control (i.e., a range of scores on the measure), whereas Rawal and Rice (2012b) defined low executive control as scores falling one standard deviation below the mean on a block design task. As we noted previously, the block design task employed in the Rawal and Rice (2012b) study is more readily known as a measure of visuo-constructional ability than as the executive control task for which the authors proposed to use it, and therefore it is difficult to confidently conclude that the block design task taps into executive control per se. Notably, neither Hitchcock et al. (2014a) nor Rawal and Rice (2012b) investigated the subcomponents of rumination, and the present study is the first, to the authors’ knowledge, to examine the interactive relation between rumination and executive control when processing emotional and nonemotional information and their effects on OGM in adolescence.

## Study aims

In this study we aimed to investigate two mechanisms of the CaR-FA-X model and their interacting effects in a nonclinical adolescent population. Previous research, particularly studies conducted with adults, had suggested that rumination and executive control may be underlying factors in OGM, and therefore in the present study we examined this relationship in an adolescent population. We investigated whether the relationship between rumination and executive control impacts OGM differently, depending on the subcomponents of rumination or the emotional stimuli used in the cognitive task. Because reflective pondering has been associated with reduced symptoms of depression and more coping in adolescence, reflective pondering may serve as an adaptive factor in memory retrieval. A greater understanding of the theoretical foundations of OGM could provide an opportunity for refinement to models of OGM, particularly with a focus on adolescent populations. We examined the prospective relationship between two mechanisms of the CaR-FA-X model in an adolescent sample between 13 and 16 years of age. Further, we assessed the two subcomponents of rumination, brooding rumination and reflective pondering, by employing emotional and nonemotional tests of executive control. These mechanisms were investigated both in isolation and in interaction to predict OGM at a 6-month follow-up. Using a longitudinal follow-up design, we predicted that:

1. Executive control for emotional information would predict OGM;
2. Brooding rumination would predict OGM;
3. Reflective rumination would predict fewer instances of OGM;
4. Executive control for emotional information and brooding rumination would interact to predict OGM; and
5. Executive control for emotional information would interact with reflective pondering to predict fewer instances of OGM.

## Method

### Participants

The sample consisted of 149 secondary school adolescents (37% male and 64% female) between 13 and 16 years old (Wave 1 [W1]: *M* = 13.85, *SD* = 0.78; Wave 2 [W2]: *M* = 14.28, *SD* = 0.88), recruited from four schools in Scotland, U.K. At the first data collection point, the pupils were in Secondary Year 2 (S2), Secondary Year 3 (S3), Secondary Year 4 (S4), or Secondary Year 5 (S5). At the second data collection point, all pupils had moved up a year (i.e., S2 became S3, S3 became S4, and so on). Ethical approval was obtained for the study, and each parent or guardian provided written consent. Written assent was provided by each adolescent for both waves (W1 and W2) of the study. All adolescents with appropriate parental consent and written assent participated. No exclusion criterion was applied at recruitment, which was similar to other studies (e.g., Gathercole, Pickering, Ambridge, & Wearing, 2004). Uptake of free school meals in participating and nonparticipating schools was recorded as a proxy measure of SES of the school catchment area. The uptake of free school meals, in this regard, has been used as a factor signifying socio-economic disadvantage and deprivation (Hobbs & Vignoles, 2010). The average percentage of uptake of free school means in Scotland is 10% (range = 0%–42%; School Meals Data set, 2013). In the present sample, the uptake of free school meals was 16% (range = 6%–20%), suggesting that the schools included in the present study were in the moderate range of socio-economic deprivation. Among the 81 secondary schools asked to take part in the project, there were no differences in the uptake of free school meals in the participating versus nonparticipating schools, *t*(79) = – 1.58, *p* = .12. Participants were assessed at two time points, 6 months apart. Thirteen participants (8.8% attrition) from W1 did not complete the second testing session. Scheduling difficulties, early graduation, and personal family issues were the main causes of attrition.

### Procedure

Data were collected during school hours at each participant’s school. In both waves of the study, adolescents were individually administered a sequence of tasks that included the minimal instruction autobiographical memory test (AMT), an emotional and nonemotional computerized executive control task, a measure of reflective pondering and brooding rumination, and self-report questionnaires of depressive and anxiety symptoms. At both time points, the executive control task and the AMT were counterbalanced to control for order effects and fatigue. To reduce mood-priming effects, the rumination, anxiety, and depression measures were administered last and were also counterbalanced using a random number generator.

### Measures

#### Autobiographical memory: The minimal-instruction autobiographical memory test (MI-AMT; Debeer, Raes, & Hermans, 2009)

The MI-AMT was employed as a measure of autobiographical memory. The MI-AMT is a cued recall methodology in which individuals are asked to respond with memories to valenced cue words within a given time frame. Unlike in the traditional AMT, we asked participants to generate memories within 60 seconds in response to cue words without stating that these memories should be specific. Research with adults has suggested that the original AMT (Williams & Broadbent, 1986) may not be sensitive enough to assess memory specificity in nonclinical samples (Debeer et al., 2009). Participants were asked, “Can you write down an event that the word ____ reminds you of?” The instructions and cue words were read aloud to participants and presented visually (i.e., written on an A4 sheet of paper). The instructions stated that memories must be older than 1 week and that the same memory should not be given more than once. No examples and no practice words were provided. Two word sets containing 12 cue words were taken from previous OGM research with adolescents (Rawal & Rice, 2012b; W1 positive word set: *loyal*, *joy*, *smile*, *achieve*, *loved*, *ambitious*; W1 negative word set: *mistake*, *rejected*, *weakness*, *needy*, *angry*, *tired*; W2 positive word set: *friendly*, *happy*, *respect*, *caring*, *sunny*, *perfect*; W2 negative word set: *failure*, *disliked*, *ugly*, *useless*, *worse*, *lonely*). OGM was measured as a single, unidimensional trait for positive and negative words combined (see Griffith et al., 2009). The cue words were counterbalanced in each set and matched for emotionality, imageability, and word frequency.

Responses were coded by the first author (25% were double-coded by a trained independent researcher) as specific (memory of a particular event that occurred at a particular time and place, within one day), categoric (memory of a number or category of events; e.g., the weekends), extended (memory that lasted more than a day; e.g., the school summer holidays), a semantic associate (general semantic information that is not a memory; e.g., my mum), an omission (no memory provided), or part of a rest category (incomplete answers, not in line with the instructions) as per a narrative analysis of each memory.^1^ The number of overgeneral memories (categoric and extended) was the dependent variable used in the analysis. Interrater reliability was high at both time points. The raters agreed on 96.9% of the responses at baseline (*κ*_W1_ = .92) and 90.2% of the responses at the second time point (*κ*_W2_ = .86). The MI-AMT has shown good interrater reliability in previous studies with adolescent populations: *κ* = .87 in Heron et al. (2012), *κ* = .78 in Griffith et al. (2009), and *κ* = .92 in Smets, Griffith, Wessel, Walschaerts, and Raes (2013). The AMT showed good internal consistency at the first (*α*_W1_ = .72) and second (*α*_W2_ = .76) time points, as well as modest test–retest reliability (*r* = .43, *p* < .001).

#### Executive control: Internal switch task (IST; De Lissnyder, Koster, Everaert, et al., 2012)

The IST is a valenced measure of executive control for internally represented information. Researchers have distinguished between external and internal processing (Chun, Golomb, & Turk-Browne, 2011). *External processing* refers to processing information from the external world, whereas *internal processing* refers to the processing of information held in working memory. Previous research has suggested that measuring executive control for externally represented information may not be the most beneficial way to either measure executive control or uncover any relationship between executive control and rumination (De Lissnyder, Koster, & De Raedt, 2012; De Lissnyder, Koster, Goubert, et al., 2012). Because rumination is characterized by repetitive, internal negative thoughts and executive control requires internal processing, a task that taps into this internal process may be more effective in detecting a relationship between rumination, executive control, and OGM. The IST measures executive control when processing information that is internally represented. Importantly, this task measures executive control when processing emotional and nonemotional information and primarily taps into switching ability, as well as working memory and inhibitory processing, providing a top-down measure of executive control.

**Fig. 2:**
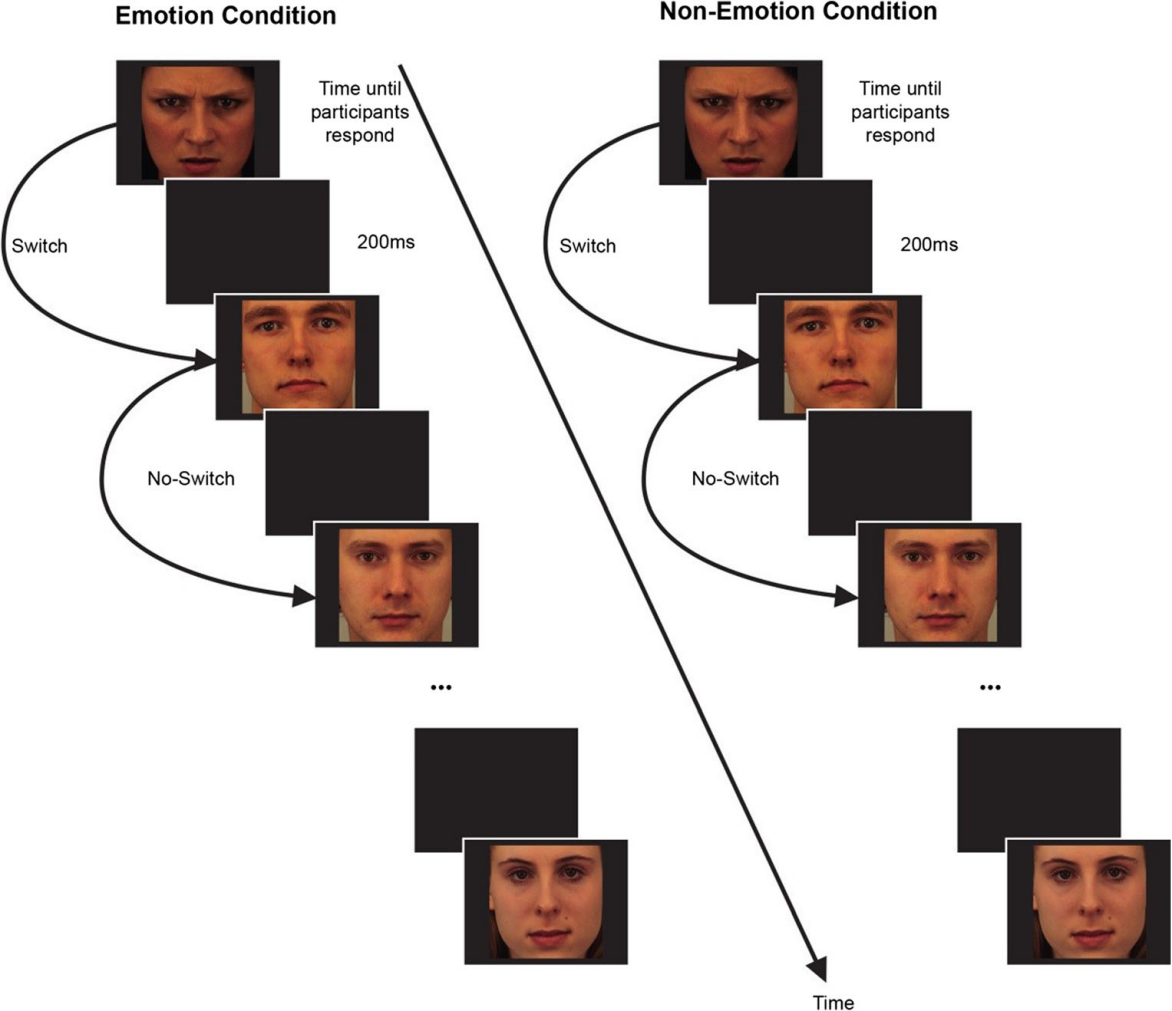
An example of a block of trials within each condition during the internal shift task (constructed by author based on an image from De Lissnyder, Koster, Everaert, et al., 2012)

The IST is a computer-based task, programmed in E-Prime to measure executive control for emotional and nonemotional information. Forty-eight (24 angry, 24 neutral) faces taken from the Karolinska Directed Emotional Faces (KDEF; Lundqvist, Flykt, & Öhman, 1998) were presented on a computer screen, one at a time. The task consisted of two counterbalanced conditions (emotion and nonemotion) and 24 blocks of trials (12 blocks in each condition), with each block contained 10–14 randomized faces (see Fig. 2). Three practice blocks were given before the start of each condition. The faces appeared at an intertrial interval of 200 ms, and the orders of trials and faces were randomly determined with a replacement procedure. The participant’s task was to keep a mental count of the number of faces that appeared on the screen, depending on the task condition (i.e., the number of male faces and the number of female faces, or the number of angry faces and the number of neutral faces). Participants were asked to press the spacebar as fast as possible to indicate that they had updated their mental count, which measured their reaction time in response to switching and updating their mental count. To ensure consistent counting, participants used the number pad on the keyboard to indicate how many faces they had counted within each block of trials.

Switch costs were calculated as the reaction time difference between switch and no-switch trials and were used as the main unit of analysis (switch: male–female, female–male, angry–neutral, neutral–angry; no-switch: male–male, female–female, angry–angry, neutral–neutral). Executive control was operationalized as the range of reaction time scores on the IST. Higher reaction times (i.e., greater switch costs) reflected lower levels of executive control, and lower reaction times (i.e., lower switch costs) reflected greater levels of executive control. There were no cutoff scores to define the high- and low-impairment groups; instead, the range of scores across the task was used (similar to previous research with nonclinical adolescent populations; Hitchcock et al., 2014a). To reduce the statistical influence of outliers, median scores were used (De Lissnyder, Koster, Everaert, et al., 2012). In line with previous research, both correct and incorrect trials were included in the analysis (De Lissnyder, Koster, Everaert, et al., 2012). Internal consistency was excellent within both the emotion condition (*α*_W1_ = .80, *α*_W2_ = .80) and the nonemotion condition (*α*_W1_ = .81, *α*_W2_ = .77). The task displayed modest test–retest reliability in both the emotion condition (*r* = .48, *p* < .001) and the nonemotion condition (*r* = .38, *p* < .01).

#### Rumination: Ruminative Response Scale of the Response Style Questionnaire Rumination (RRS; Nolen-Hoeksema, 1991)

Based on a 4-point Likert scale (1 = *almost never*, 2 = *sometimes*, 3 = *often*, 4 = *almost always*), the RRS is a 21-item self-report questionnaire that measures ruminative responses. Higher scores are indicative of a greater ruminative tendency. The brooding rumination and reflective pondering subscales were extracted from the RRS, following the work of Treynor et al. (2003). Internal consistency was excellent for both brooding rumination (*α*_W1_ = .79, *α*_W2_ = .85) and reflective pondering (*α*_W1_ = .77, *α*_W2_ = .80). The task also displayed good test–retest reliability for both the brooding scale (*r* = .68, *p* < .001) and the reflective pondering scale (*r* = .66, *p* < .001). Similar psychometric properties have been reported with adolescent populations. Cox, Funasaki, Smith, and Mezulis (2012), for example, reported internal consistencies of *α* = .71 for the reflection subscale and *α* = .79 for the brooding subscale in their sample.

#### Depression: The Beck Depression Inventory–II (BDI-II; Beck, Steer, Ball, & Ranieri, 1996)

The BDI-II is a 21-item self-report measure of depressive symptom severity (each item ranging from 0 to 3), developed to measure symptoms of depression in adolescent (13 years of age and over) and adult samples. Scores are summed to calculate a total BDI-II score, which can range from 0 to 63. As per previous research (Balazs et al., 2013: Basner et al., 2014; Osman, Kooper, Guttierez, Barrios, & Bagge, 2004; Wisco & Nolen-Hoeksema, 2010), we removed the sex and suicidal ideation questions from the scale as these were considered unsuitable for the age of the sample and for the pseudo-anonymous nature of the prospective study design. Given the omission of two questions, the scale ranged from 0 to 57 in severity in the present sample. Lanteigne (2012) omitted suicidal ideation and the loss of interest in sex in research with a community school sample of adolescents and reported high internal consistency for the 19-item version of the BDI-II (*α* = .84). The internal consistency in the present study was excellent (*α*_W1_ = .94, *α*_W2_ = .94), as was test–retest reliability (*r* = .83, *p* < .001).

#### Anxiety: Multidimensional Anxiety Scale for Children 2nd edition (MASC-II; March, Parker, Sullivan, Stallings, & Conners, 1997)

The MASC-II is a 50-item (each item ranging from 0 to 3; *never*, *rarely*, *sometimes*, and *often true about me*) self-report measure of anxiety. Rated on a 4-point Likert-type scale, higher scores are indicative of greater anxiety. Scores are summed from specific subscales and items to calculate a total MASC-II score, which can range from 0 to 150. Excellent Cronbach’s alpha values were found for the MASC-II scores (*α*_W1_ = .94 and *α*_W2_ = .94), as well as good test–retest reliability (*r* = .82, *p* < .001). The MASC-II has demonstrated good internal consistency (March et al., 1997), particularly in nonclinical adolescent samples (Muris, Merckelbach, Ollendick, King, & Bogie, 2002), and good test–retest reliability at a 3-month interval (*r* = .87; March et al., 1997). The MASC-II correlates significantly (*r* = .63) with other anxiety measures but nonsignificantly (*r* = .19) with measures of depression, and it has been shown to discriminate anxious children and adolescents from the general population as well as from other clinically diagnosed populations (March et al., 1997).

### Analytical strategy

The data were screened for skew and missing data scores. All measures displayed skewness and kurtosis values between – 1.0 and + 1.0, suggesting that no transformation of the data was necessary. Two participants’ data had Mahalanobis *D*^2^ values of <.001 and were excluded from all further analyses. At baseline, 82.99% had a complete data set, and 74.15% had a complete data set at W2. Missing data were identified on the depression and anxiety questionnaires at both time points (individual items: from 0.7% to 2%, *n* = 1–3). Data were missing completely at random (Little’s MCAR; *χ*^2^ = 3,584.44, *df* = 3553, *p* = .35). Missing data were imputed using multiple imputation (MI) with SPSS 22. MI is effective for up to 80% missing data and provides unbiased estimates when the data are missing completely at random, as in the present data set. Imputed values matched the original values (i.e., MASC-II had a score of 0, 1, 2, or 3), and five data sets were imputed. MI allowed for analysis on 100% of the participant data. Pooled estimates were created through SPSS version 22. The estimates were averaged across all five imputed data sets when pooled estimates were not available in SPSS (see Jones, Heim, Hunter, & Ellaway, 2014).

A hierarchical multiple linear regression analysis was applied to the data. To allow the detection of a medium effect size with a power of .80 and significance of *α* < .05, an a priori power analysis indicated that 113 participants would be required (Tabachnick & Fidell, 2007). Step 1 controlled for five covariates; age, gender, depressive symptoms, anxiety symptoms, and baseline autobiographical memory. Symptoms of anxiety and depression have previously been shown to impact task performance on cognitive tasks (Altamirano, Miyake, & Whitmer, 2010; Cisler & Koster, 2010). Predictor variables (brooding rumination, reflective pondering, and executive control for emotional and nonemotional information) were added at Step 2, and interaction effects between the predictor variables were added at Step 3. To control for possible multicollinearity when including interaction terms, each predictor variable was mean-centered. Finally, follow-up simple slope analyses based on Aiken and West’s (1991) standard procedure were assessed using the Hayes (2012) PROCESS macro for SPSS 21. The predictor variable (executive control for emotional information) and control variables (age, gender, depressive symptoms, anxiety symptoms, brooding rumination, and baseline autobiographical memory) were standardized prior to the moderation analysis.

## Results

All main study variable means and standard deviations are reported in Table 1. Also in Table 1 are reports of whether the scores were significantly different at W2 than at W1. We observed no significant difference between W1 and W2 overgeneral memory scores, nor between W1 and W2 executive control scores for nonemotional information, W1 and W2 depression scores, or W1 and W2 anxiety scores. Executive control for emotional information showed significant decreases at W2 in comparison to W1. Rumination, in the form of either brooding rumination or reflective pondering, significantly decreased over time. As we noted earlier, rumination can be relatively unstable in childhood (Driscoll, 2004) and may become more trait-like in adolescence (Rood et al., 2009). The age of our sample (mean age = 13 years) may therefore have impacted on these correlations, since rumination may not yet have been a stable, trait-like construct. Additionally, tests of executive function are limited by the degree to which scores are stable over time (Burgess 1997; Miyake et al., 2000). Although this issue is not readily understood in the literature, it has been suggested that the ability to exert control over cognitive functioning in a task is strongest when the task is novel (Rabbitt, 1997). Given that tasks are only novel once, this could provide an explanation for the differences from W1 to W2 in our executive control task. Future research that permitted multiple time points and longer follow-up times between sessions could clarify more specifically how and when these factors develop and change within and across development, and could provide further information about the stability of these variables during and across adolescence.

**Table 1:** Mean scores, standard deviations, and *t* -tests (with effect sizes) for all W1 and W2 measures

	Wave 1		Wave 2		*t*	*d*
	*M*	*SD*	*M*	*SD*		
Autobiographical memory: AMT-MI Overgeneral memory	4.04	2.23	4.16	2.49	– 0.56	0.05
Depressive symptoms: BDI-II	15.85^a^	11.96^b^	14.84	11.33	1.81^a^	0.30
Anxiety symptoms:: MASC-II	61.64^a^	24.28^b^	59.69^a^	25.93^b^	1.55^a^	0.26
Executive control: IST switch cost (Emotion)	541.98	310.17	485.43	293.66	2.22^*^	0.38
Executive control: IST switch cost (Nonemotion)	530.67	298.98	485.17	287.04	1.63	0.27
Rumination: RRS brooding rumination	11.36	3.57	10.74	3.67	2.58^*^	0.43
Rumination: RRS reflective pondering	9.54	3.51	8.99	3.41	2.22^*^	0.38

AMT-MI = autobiographical memory test–minimal instruction, BDI-II = Beck Depression Inventory II, MASC-II = Multidimensional Anxiety Scale for Children, IST = internal shift task, RRS = Ruminative Response Scale. ^a^Pooled estimates. ^b^Estimates averaged from results of the five imputed data sets. ^*^p < .05.

Bivariate correlations within and across time points are displayed in Table 2. Within W1 of the study at the first time point, OGM was not correlated with any other W1 predictor: brooding rumination, reflective pondering, or executive control for emotional or nonemotional information. Within W2, OGM was correlated only with W2 brooding rumination and with no other W2 variables. Across time points, W1 symptoms of anxiety and depression as well as brooding rumination and reflective pondering were correlated with W2 OGM.

**Table 2:** Bivariate pooled correlations

	2.	3.	4.	5.	6.	7.	8.	9.	10.	11.	12.	13.	14.	15.	16.
1. W2 overgeneral memory	.06	.06	.20^**^	.14^*^	.43^***^	.23^**^	.20^**^	– .08	– .07	.13	.03	.19^*^	.07	– .14	– .12
2. Age	–	– .01	.19^**^	.00	– .06	.20^**^	.22^**^	.07^*^	– .10^*^	.11	– .05	.03	.13	– .07	.05
3. Gender			.38^***^	.31^***^	– .08	.24^**^	.17^*^	– .03	.01	.32^***^	.36^***^	.25^***^	.24^***^	.16	.09
4. W1 depression symptoms			–	.69^***^	– .04	.71^***^	.58^***^	– .14^*^	– .07	.83^***^	.60^***^	.60^***^	.63^***^	– .07	.03
5. W1 anxiety symptoms				–	– .06	.65^***^	.52^***^	.05	.06	.65^***^	.82^***^	.54^***^	.57^***^	.11	.13
6. W1 overgeneral memory					–	.12	.07	.02	.06	– .09	.10	.12	– .09	– .08	– .09
7. W1 brooding rumination						–	.67^***^	– .09	– .04	.61^***^	.55^***^	.68^***^	.63^***^	– .05	– .00
8. W1 reflective pondering							**–**	– .15	.03^*^	.48^***^	.52^***^	.50^***^	.66^***^	– .16	– .09
9. W1 switch cost (emotion)								–	.44^***^	– .15	– .02	– .02	– .14	.48^***^	.45^***^
10. W1 switch cost (nonemotion)									**–**	– .07	.06	.07	– .06	.38^***^	.33^***^
11. W2 depression symptoms										–	.68^***^	.69^***^	.68^***^	– .03	.05
12. W2 anxiety symptoms											–	.62^***^	.65^***^	.06	.04
13. W2 brooding rumination												–	.65^***^	– .06	.01
14. W2 reflective pondering													–	– .12	.02
15. W2 switch cost (emotion)														–	.41^***^
16. W2 switch cost (nonemotion)															**–**

^***^p < .001, ^**^p < .01, ^*^p < .05.

A hierarchical multiple regression analysis was conducted to assess whether brooding rumination, reflective pondering, or executive control for emotional or nonemotional information (measured by larger switch costs) would prospectively predict overgeneral memory. Average standardized betas (computed by summing the standardized coefficient estimates across results from the five imputed data sets and dividing by five) are reported in Table 3. Across the five imputed data sets, all standardized betas were identical. The final regression model, including all main effects and interaction terms, was significant, *F*(13, 133) = 4.16, *p* < .001, and explained 29% of the variance. Model 1 was significant at *p* < .001, Model 2 was significant at *p* < .001, and Model 3 was significant at *p* < .001. All variance inflation factors were <3, indicating no problems with multicollinearity.

**Table 3:** Hierarchical regression analysis of the effects of W1 scores for brooding rumination, reflective pondering, and executive control for emotion and nonemotion on W2 overgeneral memory scores

Step	Predictors	Dependent Variable: Number of Overgeneral Memories		
		Step 1 β^***^	Step 2 β^***^	Step 3 β^***^
1.	Age	.06	.06	.07
	Sex	.02	.02	.02
	W1 Depressive symptoms	.17	.13	.12
	W1 Anxiety symptoms	.04	.05	.05
	W1 Overgeneral memory	.45^***^	.45^***^	.45^***^
2.	W1 Brooding rumination		– .01	.04
	W1 Reflective pondering		.06	– .01
	W1 Switch cost (emotion)		– .04	– .01
	W1 Switch cost (nonemotion)		– .07	– .09
3.	W1 Switch cost (emotion) * Brooding			.16
	W1 Switch cost (emotion) * Reflect			– .28^**^
	W1 Switch cost (nonemotion) * Brooding			– .14
	W1 Switch cost (nonemotion) * Reflect			.13

Pooled estimates are reported throughout table. ^***^p < .001, ^**^p < .01.

The first step of the regression accounted for a significant proportion of the variance in OGM. Almost one quarter (24%) of the variation in OGM could be accounted for by the variables entered at the first step in the regression, *F*(5, 141) = 8.82, *p* < .001. Baseline OGM was the only significant predictor of OGM at W2. Brooding rumination, reflective pondering, and switch costs for emotion and nonemotional information were added at Step 2. This step was nonsignificant, so once again only baseline OGM was a significant predictor, *F*_change_(4, 137) = 0.45, *p* = .775. Thus, we found no evidence that brooding rumination, reflective pondering, or switch costs for the emotion and nonemotion conditions individually predicted OGM. At Step 3, the interaction variables were added. The interaction terms explained an additional 4% of the variance in OGM at W2. Although the *R*^*2*^ *change* at this step for the collective variance of these coefficients was not statistically significant, *F*_change_(4, 133) = 1.92, *p* = .111, ∆*R*^2^ = .04, reflective pondering did significantly interact with switch costs in the emotion condition to predict OGM at W2 (*β* = *–* .28, *p* = .01).^2^

To check that these results did not differ as a function of the scoring algorithm used for OGM, the analyses were rerun using the proportion of OGM as the outcome variable, with and without the inclusion of omissions, and using the number and proportion of specific memories, with and without omissions. The beta values were similar when the unit of analysis was either the proportion of overgeneral memories (*β* = – .28, *p* = .01) or the proportion of overgeneral memories minus omissions (*β* = – .24, *p* = .03). Similarly, in the opposite direction, beta values were similar for the number of specific memories (*β* = .20, *p* = .03), the proportion of specific memories (*β* = .20, *p* = .03), and the proportion of specific memories minus omissions (*β* = .19, *p* = .04).

Follow-up simple slope analyses were conducted to test whether the relation between executive control for emotional information and OGM differed at low, mean, and high levels of reflective pondering. The simple slope analyses decomposing the baseline executive control for the Emotional Information × Baseline Reflective Pondering interaction showed that when reflective pondering is high, there is a significant negative relation between executive control and OGM, *β* = – .21 (95% CI = – .41, – .01), *p* = .04. At the mean value of reflective pondering, there was a nonsignificant negative relation between executive control and OGM, *β* = .06 (95% CI = – .21, .09), *p* = .44. When reflective pondering was low, we found a nonsignificant positive relation between executive control and OGM, *β* = .09 (95% CI = – .12, .30), *p* = .38.

## Discussion

The primary aims of this study were to prospectively examine the capture and rumination and executive control mechanisms of the CaR-FA-X model and to investigate whether they independently and in interaction predicted OGM in adolescence. We found that executive control for emotional information was negatively associated with OGM, but only when reflective pondering levels were high. Thus, in the context of higher levels of reflective pondering, as switch costs increased (i.e., lower executive control) when processing emotional information, there was a decrease in OGM (i.e., greater specificity). Moreover, this relation held after controlling for baseline OGM scores and symptoms of depression and anxiety. This suggests that reflective pondering is a moderator in the executive control and OGM relationship, particularly when processing emotional material. Interestingly, brooding rumination, reflective pondering, and executive control for emotional or nonemotional information did not independently predict OGM.

Although executive control and rumination did not independently predict OGM in the present study with adolescents, rumination interacted with executive control to predict OGM over time, but not in the way that the model suggests. We did not find rumination and executive control to interact to predict greater OGM, as is proposed in the CaR-FA-X model. However, we did find that greater switch costs when processing emotional information can lead to improved specificity over time, but only in the context of high levels of reflective pondering. These results build upon the CaR-FA-X model to show that nonclinical adolescents who have difficulty in processing emotional information have greater memory specificity over time, but only when those adolescents are also high reflective ruminators.

Although a link between rumination and executive control has been highlighted in the adult (De Lissnyder, Koster, Derakshan, & De Raedt, 2010) and adolescent (Hilt, Leitzke, & Pollak, 2014) literature, no study has investigated the subcomponents of rumination and the relationship with emotional and nonemotional executive control on OGM, making it difficult to draw conclusions about the present findings. It is possible that reflective ponderers were able to activate a particular skill set that allowed the search through the hierarchy for a specific memory to continue, despite demonstrating low executive control. Rumination, as per previous studies, was assessed by asking participants to state how often they engaged in a range of ruminative thinking when they felt down, sad or depressed. It did not measure active ruminative thinking but instead a person’s predisposition to ruminate. It could therefore be argued that as reflective pondering is adaptive with a more positive focus on problem solving, when asked to recall specific memories those who were high reflective ponderers were able to access a set of skills (e.g., problem solving, nonjudgmental focus on other memories in the search for a specific one, allowing to move down the hierarchy) that aided the search through the hierarchy for a specific memory. Given the finding that reflective pondering may be an adaptive factor between executive control and OGM, the CaR-FA-X model could be refined to include reflective pondering as a possible protective factor to OGM, specifically in nonclinical adolescent populations, who may present with lower executive control. Further research is warranted, however.

Contrary to previous research in adult samples (De Lissnyder, Koster, Goubert, et al., 2012; Koster, De Lissnyder, Derakhshan, & De Raedt, 2011; Romero, Vazquez, & Sanchez, 2014) brooding rumination was not associated with executive control and only correlated with OGM at W2. In the adult literature, brooding rumination but not reflective pondering has been shown to mediate the relationship between symptoms of depression and OGM (Debeer et al., 2009). Although the CaR-FA-X model does not account for the different functions of the subcomponents of rumination, a possible explanation for the present null finding in comparison to the adult literature is the age of our sample. The mean age of our sample was 13 years old and it may be that executive control was not yet impaired enough or present for long enough to have an effect to a degree that would be reflected in higher levels of brooding rumination and subsequently OGM. The lack of relationship between brooding rumination and OGM has also been reported in previous studies with community adolescent samples. Across two studies, Schoofs et al. (2012) found no association between brooding rumination and OGM. It could be that brooding rumination is only associated with OGM in clinical populations, or in nonclinical adult populations. Indeed, as the present sample increased in age (from W1 to W2) brooding rumination at W1 was significantly correlated with OGM at W2, despite no correlation at W1. Moreover, W1 anxiety, depression, and rumination were also correlated to W2 OGM further suggesting age related differences. However, given the correlational nature of these findings we are not able to infer causality. Furthermore, studies with child and adolescent populations are lacking and research that compare the developmental trajectory across childhood and adolescence with adult populations on the subcomponents of rumination and their relation to OGM have yet to be conducted.

Employing a sample of community adolescents, our findings build on previous work with “at risk” adolescent populations. Rawal and Rice (2012b) reported an interaction between rumination and visuo-constructional ability such that high rumination in the context of low visuo-constructional ability predicted reduced AM specificity 1 year later. Our findings add nuance to this documented effect. Although we did not find brooding rumination to interact with executive control to predict OGM, we did find that reflective pondering, a subcomponent of rumination interacted with executive control to predict reduced OGM over time. These findings contradict Hitchcock et al. (2014a), who reported no interaction between rumination and executive control. This may be because Hitchcock et al. (2014a) included a sample of trauma-exposed adolescents or because they did not investigate the subcomponents of rumination. Taken together, our findings suggest that rumination and executive control interact to predict reduced OGM in adolescents. This relationship had a different effect on OGM depending on the type of rumination. Further research will be warranted to explore this relationship further.

Interestingly, the present findings and those of Rawal and Rice (2012b) and Hitchcock et al. (2014a) do not support the theoretical perspective that the mechanisms of the CaR-FA-X model can work in isolation to predict OGM in a community adolescent population. Although cross-sectional research has shown OGM to be associated with inhibitory processing in nonclinical community children (Raes et al., 2010) and category fluency (Valentino et al., 2012) in clinical inpatient adolescents, all three prospective studies with child and adolescent samples (i.e., the present findings; Hitchcock et al., 2014a; Rawal & Rice, 2012b) collectively do not support the mechanisms of the CaR-FA-X model working in isolation. It is important to note that Raes et al. (2010) measured behavioral inhibition using a parental self-report version of the Early Adolescent Temperament Questionnaire. Reliance on self-report measures has been subject to criticism in the literature and Raes et al. (2010) note that future research should “obtain more objective laboratory indices of inhibitory control.” In the present study, an objective, general measure of executive control was employed with a focus on emotional and nonemotional stimuli.

There has been considerable heterogeneity between studies with regard to the aspects of executive control measured, and future research will be needed, given the limited (and conflicting; Hitchcock et al., 2014a) research in this area. Although the present findings suggest that lower executive control when processing emotional material, in the context of high levels of reflective pondering, was associated with reduced OGM over time, this was examined over a six month period. A longer follow-up could permit a more nuanced understanding of the development of OGM. Furthermore, no comment can be made regarding any adaptive (e.g., protective against the development of depression) or maladaptive outcomes (e.g., development of depression) over a longer period. It would be interesting for future research to explore whether reflective pondering impacts executive control and OGM, in turn protecting against the development of depression.

Our findings show that the relationship between reflective pondering and executive control in reducing OGM in adolescence was specific to processing emotional stimuli. The literature does tend to support the notion that rumination is closely associated with the processing of emotional information (De Lissnyder, Koster, Goubert, et al., 2012; Hilt et al., 2014). Within the depression literature, neuropsychological research findings have shown that adults with depression have a heightened attentional engagement toward negative emotional aspects of faces (Leyman, De Raedt, Schacht, & Koster, 2007). Our findings may have tapped into the emotional difficulties that characterize major depressive disorder (American Psychiatric Association, 2013). It could be that the emotional-processing difficulties reported in depression may be present in vulnerability factors associated with the disorder. At present, it remains unclear whether differences in the relationship between rumination, executive control, and OGM are due to the type of emotional information processed (i.e., sad vs. angry). A fruitful avenue for future research would be to test whether the relationship is influenced by the valence of faces presented within the executive control task. This may influence our theoretical understanding of the underpinnings of OGM.

Our study has a number of strengths. First, it used a prospective longitudinal design with participants who were at an important period of adolescence. We were able to investigate changes over a six-month period at a time at which executive processes and the prefrontal cortex are still developing. Second, our assessment of rumination allowed us to investigate the different effects of brooding rumination and reflective pondering on executive control and AM scores. Third, the objective measure of executive control provided the opportunity to measure executive control when processing emotional and nonemotional information. Finally, we controlled for symptoms of anxiety and depression as research has suggested even subclinical levels can impact task performance on executive tasks (Ansari, Derakshan, & Richards, 2008; Holmes & Pizzagalli, 2007).

In addition to the strengths of the present study, several observations and future recommendations must be noted. The novel characteristics of this study, although advantageous, require validation. Accordingly, replication of these findings and expanding on the complex relationship between the mechanisms of the CaR-FA-X model in adolescence would enhance our theoretical understanding of OGM. For this study, we employed the IST. We considered this task valuable for the present research questions, however we are aware that that this task does not allow for examination of control processes within specific aspects of executive functions (i.e., inhibition, switching and updating; Miyake et al., 2000). Future research would benefit from developing a task that allows for the measurement of executive control for internally represented emotional and nonemotional information, while measuring different aspects of executive functions. Tests of executive function and executive control are limited by their test–retest reliabilities, as noted by many authors in this area (Burgess 1997; Miyake et al., 2000). Although the internal consistency of the IST was high in the present sample, we found low to moderate test–retest reliability with the IST, similar to previous research that has employed other executive control tasks (Henry & Bettenay, 2010). It is evident that this is an issue in the field beyond the present study. However, since we are the first authors to have used the IST with adolescent populations, future research should establish the reliability and validity of this task for use in this population. It should also be noted that the functional avoidance was not examined and future research could benefit from examining the relationships between executive control, rumination, and functional avoidance.

Previous research has noted that memory for autobiographical events includes both episodic and sematic details (Brown et al., 2014; Conway & Pleydell-Pearce, 2000), with some suggesting that semantic processing may underlie episodic memory (Irish & Piguet, 2013). Future research that categorize memories, while also coding the representations for internal (episodic) and external (semantic) details may offer new insights into the nature of memories for past events. Indeed, previous research has demonstrated age-related differences in which older adults generate fewer internal and more external details in past events in comparison to younger adults (Addis, Wong, & Schacter, 2008; see Devitt, Addis, & Schacter, 2017, for a review). Examining the details of memory representations in adolescence may provide a more detailed understanding of the composition of OGM recall in this age group.

Despite the noted considerations, our findings have several important theoretical and clinical implications. We add to the CaR-FA-X model by demonstrating the adaptive nature of reflective pondering on OGM, when executive control is low. We also show that this relation is specific to processing emotional information. Given that the main finding emerging from the present study was the adaptive nature of reflective pondering, particularly in the context of low executive control when processing emotional information, interventions aimed at increasing reflective pondering may serve to reduce OGM. This study provides a novel contribution to the field and future research is warranted to confirm the adaptive nature of reflective pondering on the executive control and OGM relationship, and subsequently symptoms of depression.

## Footnotes

1 The training manual for the MI-AMT and scoring guide were provided by Dr. Jennifer Sumner (Columbia University). She was also provided guidance and advice on using the MI-AMT.

2 Although we measured OGM as a single unidimensional trait, we acknowledge that some readers may be interested in the valence of the cue words. We reran the analyses using the number of OGMs to positive and negative cue words. Beta values were similar when the unit of analysis was the number of overgeneral memories to negative cue words (*β* = – .24, *p* = .048) and the number of overgeneral memories to positive cue words (*β* = – .23, *p =* .033).
